# Capturing the full spectrum of T cell responses with spectral flow cytometry

**DOI:** 10.1093/oxfimm/iqaf011

**Published:** 2025-12-23

**Authors:** Anna Olofsson, Annika C Karlsson

**Affiliations:** Division of Clinical Microbiology, Department of Laboratory Medicine, Karolinska Institutet, Stockholm, 171 77, Sweden; Division of Clinical Microbiology, Department of Laboratory Medicine, Karolinska Institutet, Stockholm, 171 77, Sweden

**Keywords:** memory T cells, spectral flow cytometry, optimization, standardization, flow data analysis

## Abstract

Over a decade has passed since the first commercial spectral flow cytometry (SFC) instrument was introduced. Unlike conventional flow cytometers, SFC utilizes an array of detectors to capture the full emission spectrum of fluorochromes, from which composite signatures are deconvoluted using an unmixing algorithm. This allows fluorochromes with overlapping peaks to be used within the same panel, enabling panels with up to 50 parameters. As its availability increases, more immunologists are looking to incorporate SFC into their experiments. One area of research benefiting from the larger SFC panels is the characterization of rare cells, including antigen-specific T cells identified directly ex vivo using either antigen stimulation or major histocompatibility complex–peptide multimers. In this brief review, we outline some practical considerations when combining ex-vivo T cell stimulation with SFC, drawing on our transition from conventional to SFC. Key aspects include designing the experiment and panel for stimulated cells, acquiring high-quality reference controls, strategies to manage autofluorescence and an overview of the data analysis, including both manual and computational approaches.

## Graphical Abstract

**Figure:**
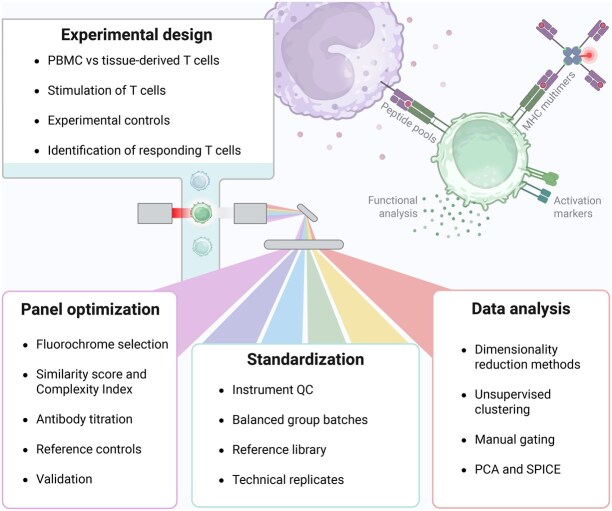

## Introduction

Since the development of the first fluoresence-based flow cytometer in the late 1960s by Dittrich and Göhde, steady technological progress has encouraged its widespread application in both research and clinical diagnostics [1]. For decades, conventional flow cytometry (CFC) dominated the field, which relies on optical filters and channel-specific detectors to capture only the peak emission signal of each flurochrome. Compensation is applied to mathematically correct for spectral overlap, a process that becomes increasingly challenging as the number of fluorochromes approaches the practical ceiling of about 28 markers, determined by the limited availability of fluorochromes with unique emission peaks.

In 2014, the first spectral flow cytometry (SFC) instrument became commercially available [2]. By utilizing an array of detectors to capture the entire emission spectrum of a fluorochrome, SFC enables the simultaneous use of fluorochromes with highly similar emission peaks but distinct off-peak signatures. Instead of compensation, it utilizes unmixing algorithms and single-stained reference controls to deconvolute the individual fluorochrome emission signatures within the composite spectrum [3]. Today, this allows for panels up to 50 parameters, with sizes expected to increase as unmixing algorithms evolve and new fluorochromes are developed [4]. In addition to increased panel flexibility, SFC enables autofluorescence (AF) to be extracted and added as an independent parameter, thereby improving resolution of samples with high AF interference. Collectively, the advantages of SFC alongside its growing availability has made it an attractive platform for researchers seeking to maximize the information gained from each sample.

Understanding what constitutes long-lasting and effective memory T cell responses is a long-standing goal of immunologists [5–14]. This pursuit is aided by flow cytometry, which is frequently used to identify and characterize antigen-specific T cells using peptide-major histocompatibility complex (MHC) multimers or by combining ex-vivo peptide stimulation with the activation-induced marker (AIM) assay and intracellular cytokine staining. Beyond basic immunology research, T cell specificity and functionality is evaluated extensively in diagnostics, infectious disease monitoring and throughout the development of vaccines, antiviral treatments and cancer immunotherapies, like checkpoint inhibitors, tumor-infiltrating lymphocytes and chimeric antigen receptor (CAR) T cells [15–21]. This brief review aims to outline some practical considerations for assessing T cell responses with SFC when transitioning from CFC.

## Experimental design

Due to their established protocols, accessibility and clinical relevance, peripheral blood mononuclear cells (PBMCs) remain the most common material when assessing human T cell responses with flow cytometry. However, the large panels and AF extraction enabled by SFC also appeal to immunologists studying tissue-derived conventional and unconventional T cells [22]. Regardless of instrument format, flow cytometry protocols and panels for tissue-derived cells need to be optimized in accordance with the tissue digestion method, cell adhesion and particularly AF. One early consideration to mitigate AF interference is to determine the AF signature of your samples before panel design and pair channels with high interference with exclusionary markers. As an example, we typically use the V7 channel (e.g. BV510) as the dump channel when designing panels based on the AF signature of PBMCs. Another important consideration regarding the material is to ensure that there are enough cells for your experiments. Unexpanded antigen-specific T cells are often rare, ranging from <100 to a few thousand cells per million PBMCs depending on the cohort and specificity. Similarly, the frequency of tissue-derived T cells is highly dependent on the tissue of origin and antigen-specificity [23]. Additionally, each donor or condition must have a negative and positive control, and some cohorts may require an unstained sample from each donor for optimal AF extraction and unmixing. In terms of the positive control, we prefer Staphylococcus Enterotoxin B (SEB) when assessing antigen-specificity in PBMC samples. This is because SEB crosslinks MHC-II and B7 to TCR and CD28 respectively, thus mimicking the peptide-TCR interaction. Furthermore, by only activating up to 30% of T cells, SEB can preserve cell viability and functionality during longer stimulations [24, 25]. However, it is ultimately the aim and material that determine the most appropriate positive control, with some other options including anti-CD3 and anti-CD28, phorbol 12-myristate 13-acetate (PMA) and ionomycin, and peptide pools activating memory T cells in most adult humans, such as peptides derived from standard vaccines or common viral infections [26, 27].

Selecting the stimulation and detection approaches are important decisions closely intertwined with the available material and experimental question. Peptide-MHC multimers (tetramers) are considered the gold standard for the identification of antigen-specific T cells, offering high specificity and compatibility with downstream analyses by requiring neither stimulation nor fixation. Tetramers are also well suited for samples with low T cell density, such as non-lymphoid tissues [23]. However, the need for prior knowledge of immunogenic peptides, donor-specific MHC alleles and peptide-MHC pairs limit their application to well defined pathogens and populations. Moreover, because of the often low frequency of antigen-specific CD4+ T cells and the lower functional avidity between the peptide-MHC class II molecule and the T cell receptor complex, tetramer-based studies typically use MHC class I tetramers and focus on antigen-specific CD8+ T cells. Conversely, stimulation by peptide pools can capture both antigen-specific CD8+ and CD4+ T cells, with shorter (8–9 amino acids) and longer (15–20 amino acids) peptides biased for the two compartments, respectively. By combining predicted or identified peptides for MHC class I and class II, it is possible to capture the immunity in most individuals independent on ethnical background [26, 28]. Peptide pools can also be used independently of known MHC–peptide combinations, allowing for an even wider range of donors and T cell specificities to be studied. In these cases, peptide pools often consist of a number of peptides (typically 15–20 amino acids long) overlapping each other by a set number of amino acids (e.g. 10 amino acids), meaning that each peptide shares part of its sequence with the next peptide. Pools with overlapping peptides generally span an entire protein or the immunogenic part of a protein, and can be used to identify new immunogenic peptides [26, 27, 29–32].

Following peptide stimulation, antigen-specific or activated T cells are identified using intracellular cytokine staining or the AIM assay, which has been reviewed and described in-depth elsewhere [33, 34]. Compared to tetramers, peptide pools together with the AIM assay capture a larger portion of the antigen-specific T cell repertoire, though cross-reactivity and TCR-independent activation may constitute a portion of the response [27, 35]. Since the AIM assay and cytokine detection rely on T cell activation, factors influencing activation and the consecutive response can affect assay sensitivity. This includes intrinsic sample characteristics, such as T-cell exhaustion or antigen-presentation capacity, as well as protocol-related factors, like the reduced functional output observed when high-affinity T cells are stimulated with higher peptide concentrations [36–38]. Furthermore, because activation markers and cytokines have distinct kinetics and cell specificities, the stimulation length, stimuli and target cells (CD8+ or CD4+ T cells) constitute key considerations when designing an AIM assay. Typically, the co-expression of two activation markers is sufficient to define antigen-specific T cells, but combining multiple markers can increase sensitivity [39]. While the AIM assay is primarily used for PBMCs, it is also a viable option for tissue-derived T cells, though the activation marker CD69 should be avoided as it is constitutively expressed by tissue-resident T cells [40, 41].

Both peptide-MHC multimers and the AIM assay are often combined with intracellular staining of granzymes, transcription factors and cytokines after stimulation, enabling further phenotypical and functional analysis of the responding T cells [27, 42–44]. Such markers may infer the strength of the response, cell maturity and the phenotype of CD8+ T cells and CD4+ T helper cell subsets.

## Panel design and optimization

When designing a SFC panel, the same principles apply as when designing a CFC panel, including reserving bright fluorochromes for low-abundance targets, and vice versa. Similarly, spreading errors and spillover are ever present challenges in flow cytometry, supporting the sustained recommendation to avoid pairing co-expressed markers with fluorochromes that have overlapping emission spectra also for SFC [45]. The spectral similarity between two fluorochromes is given as a similarity score, which ranges from dissimilar (0) to identical (1). Higher similarity scores are more challenging to unmix and scores above 0.9 are broadly considered incompatible. However, highly similar fluorochromes, such as FITC and BB515, or APC and AF647, can still be used within the same panel if preceded by thoughtful panel design and optimization. Similarity scores are collectively quantified into a Complexity index, which is a theoretical value of a panel’s overall unmixing difficulty. A low Complexity Index indicates a higher likelihood of good resolution and a lower risk of data spread, but the value will inevitably increase as more fluorochromes are added to a panel. When possible, spreading fluorochromes between cell types will simplify the panel design process and benefit performance, but we (unpublished) and others have successfully used panels exceeding 35 makers designed for T cell phenotyping, showing that stable high-parameter SFC panels are still achievable within one cell type [17, 46]. There are several online tools, like FluoroFinder, Cytek Cloud, BD Research Cloud and Miltenyi flow cytometry panel builder, available to compare the effects of different fluorochrome combinations and identify those that best suit your panel.

Antibody titration is a critical step during panel optimization in that appropriate antibody concentrations will reduce both the cost and sample background noise. In our experience, many antibodies can be used at lower concentrations with SFC, compared to CFC, meaning that re-titration of previously used antibodies is recommended. When titrating functional markers, ensure that the chosen titration is sufficient to account for sample variability by assessing and validating the titration across both strong and weak stimuli. A too low concentration will underestimate the strong T cell responses and increase variability, while an excess can promote false positive signals or conceal weaker T cell responses behind a high background. Also consider that stimulation can affect binding efficiency to the target protein, for example through receptor internalization (e.g. CD40L) or changes to the protein structure (e.g. perforin), and that degranulation obstruct intracellular detection of cytotoxicity markers [47, 48]. Solutions to these problems include anti-CD40, Golgi inhibitors (brefeldin A and/or monensin) and intracellular staining, intentional clone selection and surface staining of degranulation markers, such as CD107a [49–51]. This underlines the importance of validating the protocol both experimentally and against literature for the best and most accurate results.

The importance of high-quality reference controls in SFC cannot be understated, they build the foundation to successful unmixing and robust results. Unmixing may be performed with a combination of single-stained cells and beads, where some markers and fluorochromes are preferably unmixed with one or the other. Beads are particularly useful for lowly expressed markers with inconsistent cell-derived signals. We recommend preparing single-stain controls from both cells and beads to test for the combination that produce the best unmixing results in your panel. As with compensation controls, reference controls must be prepared in the same manner as the samples, including cryopreservation, stimulation, brefeldin/monensin, staining buffers and fixation steps, if those are present. Keep in mind that these processes may also alter any AF signatures and should therefore be applied to any unstained controls used for AF extraction. Importantly, we caution against using the positive control stimuli when recording reference controls for activation or functional markers. We found that the strong activation induced by such controls can alter the AF signature enough to disrupt the unmixing algorithm in conditions with lower activation levels. As alternatives, we recommend using beads, more representative experimental conditions, or a stimulated, unstained control. If AF interference is still present, SFC enables the extraction and inclusion of one or multiple AF signatures as parameters for unmixing. Although the AF of lymphocytes is relatively low, we found that multiple AF extraction significantly improved the resolution of markers with emission spectra overlapping the AF signals [52]. Lastly, a new reference control should be recorded when a new antibody lot is used, especially for tandem dies [45]. Fortunately, SFC allows for individual reference controls to be updated without the need of re-recording all markers in a panel, making this a less time-consuming task compared to CFC. System issues or instrument maintenance still warrant reacquisition of all reference controls, however.

Fluorescence-minus-one (FMO) controls are strongly recommended during panel optimization for difficult markers with non-discrete staining patterns. FMO controls include all antibodies in the panel except one, providing a measure of how AF, spillover, and spreading errors contribute to the signal of the omitted marker. Consequently, FMO controls serve as valuable tools when determining the gating of negative and positive populations for markers expressed on a continuum or inconsistently. Since naïve T cells lack effector functions, they can be used as an internal negative control when gating activation and functional markers after short-term stimulations [53]. However, this method fails to account for marker-specific background or the culminative background present in activated effector or memory T cells, and should therefore only be used as a complement to proper FMO controls.

Before finalizing the panel, it should be validated across a range of samples to ensure that the unmixing remains consistent when challenged by the interindividual variations within your cohort. Additionally, we have experienced unexpected interactions between peptide pools and certain antibody-fluorochrome pairings, further highlighting the importance of checking the panel across all experimental conditions before proceeding with the study samples.

## Data analysis

SFC experiments generate two types of FCS files, raw and unmixed files. The raw files enable repeated unmixing of the data if needed, but only unmixed files can be used for further analysis. While it is possible to add minor compensation (≤±5%) to unmixed data, its usage should be kept to a minimum and only as a last resort for isolated, smaller issues where optimization to the panel design, reference controls or unmixing process failed to resolve the problem. Similarly, there are options for sample normalization and batch correction, such as linear transformation, and approaches like CytoNorm, Harmony and others [54, 55]. Caution is advised, however, as they can have unpredictable effects on the data, including introducing artifacts or removing biological differences [53]. Therefore, it is recommended to adopt strategies that minimize batch effects throughout the study, including performing daily controls of the instrument, balancing groups across batches and maintaining a consistent reference library. We also recommend tracking instrument and staining variability by including technical replicates in each run, typically consisting of samples from a set of donors with well-established marker expression patterns. When changes are unavoidable, such as between antibody lots, panel updates or prolonged time between runs, we also include several bridging samples for direct comparison.

A wide range of tools and pipelines are available for analyzing the increasingly complex datasets generated by high-parameter panels, with only a few of the most commonly used methods highlighted here. After data cleanup, whether manually or with automated tools like PeacoQC, dimensionality reduction techniques like t-SNE and UMAP are two popular options for unsupervised data visualization [56]. These tools arrange cells into global structures based on the median fluorescent intensity (MFI) of selected markers and their similarity to neighboring cells. Both t-SNE and UMAP are frequently combined with unsupervised clustering algorithms like FlowSOM and PhenoGraph to identify distinct populations within a dataset [57, 58]. However, t-SNE, UMAP and clustering algorithms are highly sensitive to dataset variability, and require thorough quality control to address batch effects, donor variability and signal intensity differences before analysis. Consequently, any interesting clusters identified by these methods need to be validated through manual gating. Another limitation is that they are not inherently quantitative; however, CITRUS, and more recent workflows such as diffcyt and MACS, enable comparisons between groups across clusters, with the latter two offering formal quantitative statistical testing [59–61].

Although possible to apply unsupervised clustering on all events in a sample, we find that manually gating out the antigen-specific or memory T cell populations before clustering and dimensionality reduction techniques often benefits their analysis, for several reasons. First, the rarity of antigen-specific T cells calls for a substantial number of events to be acquired per sample, potentially across large cohorts and multiple conditions, which significantly increases the computing power necessary for data processing. A common method around this is downsampling, where a subset of cells is used to represent the whole sample. For rare populations, such as antigen-specific T cells, however, downsampling from all events may be counterproductive as it would exclude some antigen-specific cells from analysis. Conversely, it can be appropriate to downsample directly on manually gated antigen-specific T cells to homogenize cohorts with significant yield differences between samples, something that otherwise would skew the analysis towards the overrepresented samples. Second, antigen-specific or activated T cells tend to congregate as one cluster when projected alongside total memory T cells, effectively decreasing the resolution and ability to distinguish unique clusters within the antigen-specific population unless a secondary clustering step is performed. Although the initial resolution settings can be adjusted to accommodate this, that will also increase the total number of clusters and potentially complicate the analysis further. Third, limiting downstream analyses to only include samples that induced sufficiently strong T cell responses (further discussed below) will reduce the contribution of unspecific or weak responses in your analysis. In all, consider whether initial gating of your responding T cells would simplify, or even elevate, their unsupervised clustering and visualization.

**Figure 1.**
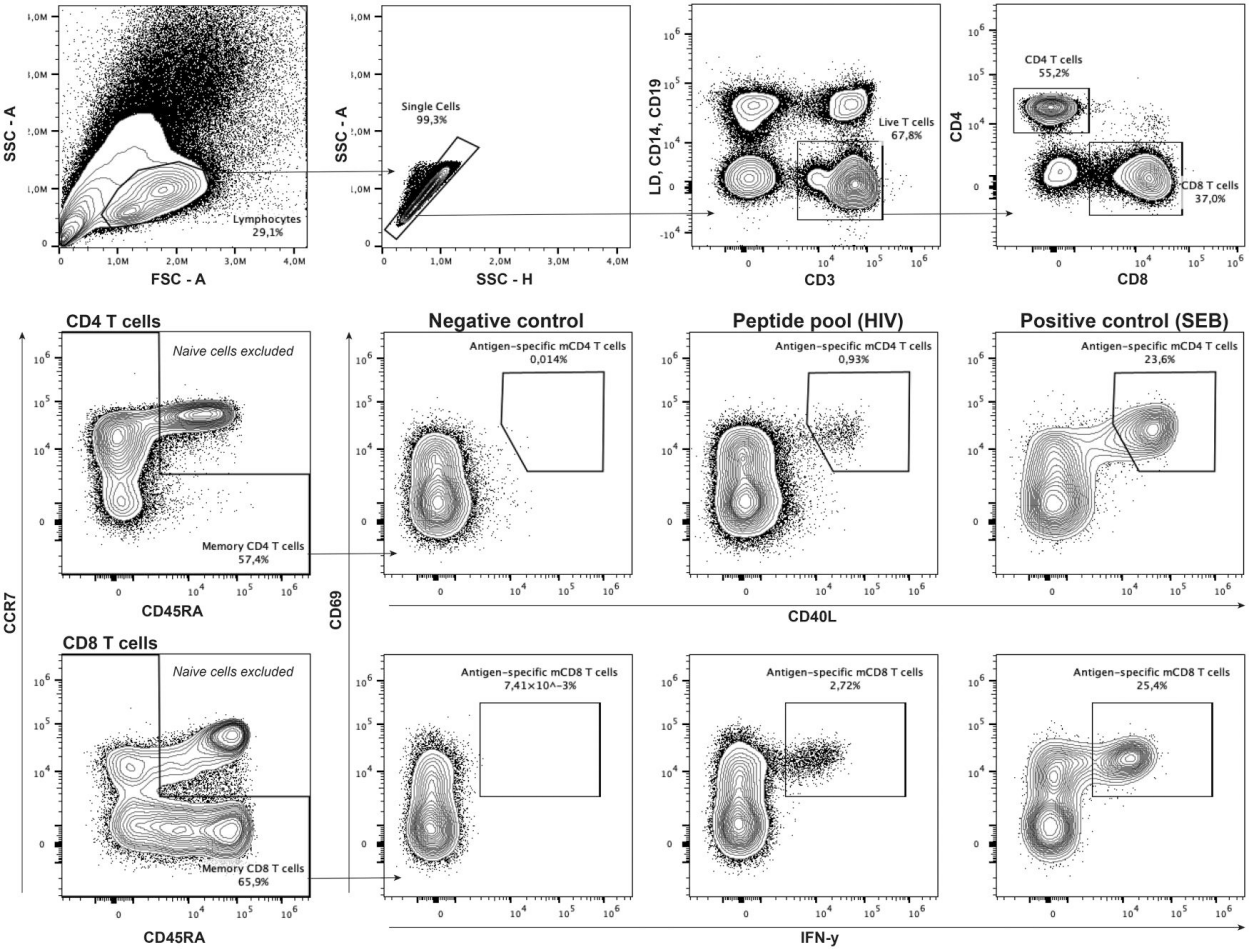
Example of gating strategy for antigen-specific memory T cells. Plots show one example of the gating strategy for antigen-specific memory T cells using the AIM assay and IFN-γ expression after peptide stimulation. Using a Dump channel containing live-dead dye (LD), anti-CD14 and anti-CD19, present dead cells, monocytes and B cells were excluded alongside the gating of CD3^+^ T cells. Naïve and memory CD4+ and CD8+ T cells are gated based on CCR7 and CD45RA expression, among which naive T cells (CCR7+CD45RA+) are excluded before the gating of antigen-specific cells from the total memory cell populations. Example data was captured on the Aurora (Cytek).

When manually gating antigen-specific T cells, start by excluding debris, dead and irrelevant cells, which includes phenotypically naïve T cells whose inability to express effector cytokines can skew downstream functional analyses (Fig. 1). The frequency of antigen-specific or responding T cells among memory T cells are then calculated as net frequency (the frequency of responding T cells after subtraction of the frequency measured in the corresponding negative control), or stimulation index (the fold change of the frequency of responding T cells relative to the corresponding negative control). Depending on the aim, it may be appropriate to define a threshold for a positive response, where only samples with responses above the threshold are included in further analyses. This allows for the exclusion of low responders whose activated cells are more likely to be driven by chance or background rather than true antigen-specificity. On the other hand, a too strict threshold risks excluding relevant antigen-specific T cells. In AIM assays, it is common to use a combination of a stimulation index score and a number of minimum cells as threshold, but it is recommended to determine an appropriate threshold based on the background level measured across your experiments [62].

In addition to unsupervised methods, there are several options to analyzing your high-parameter data using the MFI and frequencies acquired after manual gating. The dimensionality reduction method Principal Component Analysis (PCA) shows linear relationships between markers in a sample, either based on MFI or frequency. Consequently, PCA is useful to identify linear correlations between makers and to compare the variance between samples rather than individual cells. Simplified presentation of incredibly complex evaluation (SPICE) uses Boolean gating to assess the co-expression of up to six markers of interest [63]. This is a common approach to graph and quantify polyfunctionality of antigen-specific T cells. Since data variability and inconsistencies can be taken into consideration during manual gating, approaches like PCA and SPICE tend to be more reliable with highly variable data, compared to unsupervised approaches. However, manual gating is labour intensive, requires extensive training and, on its own, it is insufficient for capturing the full complexity of high-parameter panels. Nevertheless, manual gating is an indispensable step during the analysis and validation stages of any flow cytometry workflow.

Lastly, it is important to remember that neither manual gating nor unsupervised approaches avoid subjective influence. With manual gating it lies with the placement of the gates, whereas unsupervised clustering depends on the resolution chosen by the researcher. This emphasizes the need for continued efforts to standardize the field and to openly discuss and share data in the meantime.

## Challenges and future perspective

While the emerging use of SFC opens up for larger and increasingly complex panels to characterize antigen-specific T cell responses, key challenges remain. First, although *ex-vivo* T cell stimulation is a well-established tool to assess cell behavior and infection history, it lacks the environmental context of an *in vivo* infection. Complementary approaches, including infection assays, animal models, epidemiological studies, and clinical trials, are still essential to evaluate the effectiveness of these T cell responses. Second, while powerful, SFC does not reduce the cost, training, or hands-on time associated with flow cytometry. However, new fluorochromes, standardized and automated data analysis, and ready-made antibody cocktails are some active areas of research that could further expand the use of SFC in both pre-clinical and clinical settings.

## Data Availability

Data sharing is not applicable as no new data has been presented here.
